# Scoring Systems for Predicting Mortality after Liver Transplantation

**DOI:** 10.1371/journal.pone.0107138

**Published:** 2014-09-12

**Authors:** Heng-Chih Pan, Chang-Chyi Jenq, Wei-Chen Lee, Ming-Hung Tsai, Pei-Chun Fan, Chih-Hsiang Chang, Ming-Yang Chang, Ya-Chung Tian, Cheng-Chieh Hung, Ji-Tseng Fang, Chih-Wei Yang, Yung-Chang Chen

**Affiliations:** 1 Kidney Research Center, Department of Nephrology, Chang Gung Memorial Hospital, Taipei, Taiwan; 2 Division of Gastroenterology, Chang Gung Memorial Hospital, Taipei, Taiwan; 3 Laboratory of Immunology, Department of General Surgery, Chang Gung Memorial Hospital, Taipei, Taiwan; 4 Chang Gung University College of Medicine, Taoyuan, Taiwan; University of Toledo, United States of America

## Abstract

**Background:**

Liver transplantation can prolong survival in patients with end-stage liver disease. We have proposed that the Sequential Organ Failure Assessment (SOFA) score calculated on post-transplant day 7 has a great discriminative power for predicting 1-year mortality after liver transplantation. The Chronic Liver Failure - Sequential Organ Failure Assessment (CLIF-SOFA) score, a modified SOFA score, is a newly developed scoring system exclusively for patients with end-stage liver disease. This study was designed to compare the CLIF-SOFA score with other main scoring systems in outcome prediction for liver transplant patients.

**Methods:**

We retrospectively reviewed medical records of 323 patients who had received liver transplants in a tertiary care university hospital from October 2002 to December 2010. Demographic parameters and clinical characteristic variables were recorded on the first day of admission before transplantation and on post-transplantation days 1, 3, 7, and 14.

**Results:**

The overall 1-year survival rate was 78.3% (253/323). Liver diseases were mostly attributed to hepatitis B virus infection (34%). The CLIF-SOFA score had better discriminatory power than the Child-Pugh points, Model for End-Stage Liver Disease (MELD) score, RIFLE (risk of renal dysfunction, injury to the kidney, failure of the kidney, loss of kidney function, and end-stage kidney disease) criteria, and SOFA score. The AUROC curves were highest for CLIF-SOFA score on post-liver transplant day 7 for predicting 1-year mortality. The cumulative survival rates differed significantly for patients with a CLIF-SOFA score ≤8 and those with a CLIF-SOFA score >8 on post-liver transplant day 7.

**Conclusion:**

The CLIF-SOFA score can increase the prediction accuracy of prognosis after transplantation. Moreover, the CLIF-SOFA score on post-transplantation day 7 had the best discriminative power for predicting 1-year mortality after liver transplantation.

## Introduction

Liver transplantation is a viable treatment option for patients with end-stage liver disease, hepatocellular carcinoma, and fulminant hepatitis. [1]–[7] Over the past several decades, the immunosuppression, surgical techniques, and experience in managing liver allograft recipients has gradually matured and the outcome of liver transplantation has greatly improved. [8] However, organ shortage has been a new challenge because of a greater treatment demand. The selection of an adequate transplant candidate is important and the decision-making process for allocation of restricted medical resources is complex and difficult. Clinicians and investigators have, therefore, been persistently looking for objective scoring systems capable of providing accurate information on disease severity and predicting post-transplant prognosis. Main scoring systems such as the Child-Pugh score, the model for end-stage liver disease (MELD) score, the RIFLE (risk of renal dysfunction, injury to the kidney, failure of the kidney, loss of kidney function, and end-stage kidney disease) criteria, and the sequential organ failure assessment (SOFA) score, have been applied to predict the outcome after liver transplant.

In our previous report, we had compared the above main scoring systems and documented that the SOFA score calculated on post-transplant day 7 had a greater discriminative power for predicting 3-month and 1-year mortality after liver transplantation. [9] However, the SOFA score was developed from a general ICU population rather than patients with end-stage liver disease. In 2009, a group of European investigators decided to create the Chronic Liver Failure (CLIF) consortium, which was dedicated to the study of the complication of cirrhosis. The investigators used a modified SOFA score for diagnosis of organ failure, the so-called CLIF-SOFA score. Like the original SOFA score, the CLIF-SOFA score assessed the six organ systems, but it also took into account some specificities of end-stage liver disease (Table 1). [10] The purpose of this investigation was to compare the efficacy of the newly developed CLIF-SOFA score with that of commonly used scoring systems in predicting prognosis after liver transplantation.

**Table 1:** The sequential organ failure assessment (SOFA) and chronic liver failure (CLIF)-SOFA scores.

SOFA Score	0		1	2	3		4	
**Respiration**								
PaO2/FiO2	>400		>300–≤400	>200–≤300	>100–≤200 with ventilator		≤100 with ventilator	
**Coagulation**								
Platelets, ×10^3^/mm^3^	>150		>100–≤150	>50–≤100	>20–≤50		≤20	
**Liver**								
Bilirubin, mg/dL (µmol/L)	<1.2 (<20)		≥1.2–<2.0 (20–32)	≥2.0–<6.0 (33–101)	≥6.0–<12.0 (102–204)		≥12.0 (>204)	
**Cardiovascular**								
Hypotension	MAP≥70 mm Hg		MAP<70 mm Hg	Dopamine ≤5 or dobutamine(any dose)*	Dopamine >5 or epi≤0.1 or norepi ≤0.1*		Dopamine >15 or epi >0.1 or norepi >0.1*	
**CNS**								
Glasgow Coma Score	15		13–14	10–12	6–9		<6	
**Renal**								
Creatinine, mg/dL (µmol/L) or urine output	<1.2 (<110)		≥1.2–<2.0 (110–170)	≥2.0–<3.5 (171–299)	≥3.5–<5.0 (300–440)or <500 mL/day		≥5.0 (>440) or <200 mL/day	
**CLIF-SOFA Score**	**0**		**1**	**2**	**3**		**4**	
**Respiration**								
PaO2/FiO2 or Sp O2/FiO2	>400>512		>300–≤400>357–≤512	>200–≤300>214–≤357	>100–≤200>89–≤214		≤100≤89	
**Coagulation**								
INR	<1.1		≥1.1–<1.25	≥1.25–<1.5	≥1.5–<2.5		≥2.5 or platelet ≤20	
**Liver**	Same as SOFA							
**Cardiovascular**								
Hypotension	MAP≥70 mm Hg	MAP<70 mm Hg		Dopamine ≤5 or dobutamine(any dose)* or terlipressin		Dopamine >5 or epi≤0.1 or norepi ≤0.1*		Dopamine >15 or epi >0.1 or norepi >0.1*
**CNS**								
HE grade	No HE	I		II		III		IV
**Renal**								
Creatinine, mg/dL	<1.2	≥1.2–<2.0		≥2.0–<3.5		≥3.5–<5.0 or use of RRT		≥5.0
**CLIF-C OF Score**		**1**		**2**		**3**		
**Respiration**								
PaO2/FiO2 or SpO2/FiO2		>300>357		>200–≤300>214-≤357		≤200**≤214**		
**Coagulation**								
INR		<2.0		≥2.0–<2.5		≥2.5		
**Liver**								
Bilirubin, mg/dL		<6.0		≥6.0–<12.0		≥12.0		
**Cardiovascular**								
Hypotension		MAP≥70 mm Hg		MAP<70 mm Hg		Use of vasopressors		
**CNS**								
HE grade		No HE		I–II		III–IV		
**Renal**								
Creatinine, mg/dL		<2.0		≥2.0–<3.5		≥3.5 or use of RRT		

*Abbreviations: CNS, central nervous system; CLIF-C OF: chronic liver failure-consortium organ failure; CLIF-SOFA: chronic liver failure - sequential organ failure assessment; epi, epinephrine; FiO2, fractional inspired oxygen; HE, hepatic encephalopathy; INR, international normalized ratio; MAP, mean arterial pressure; norepi, norepinephrine; PaO2, arterial oxygen tension; RRT, renal replacement therapy; SOFA: sequential organ failure assessment; SpO2, pulse oximetric saturation.

## Materials and Methods

### Ethics statement

The protocol for this clinical study was designed in full compliance with the ethical principles of the Declaration of Helsinki and was consistent with Good Clinical Practice guidelines and with applicable local regulatory requirements. Because this study examined only preexisting data, written informed consent was not obtained from each patient. In its place, we informed patients of their right to refuse enrolment via telephone interview. These procedures for informed consent and enrolment are in accordance with the detailed regulations regarding informed consent described in the guidelines. This study, including the procedure for enrolment, was approved by the Institutional Review Board of Chang Gung Memorial Hospital.

### Patient information and data collection

This study was conducted between October 2002 and December 2010 in a 2000-bed tertiary care referral hospital in Taiwan. In this study, we included 323 consecutive patients with end-stage liver disease patients who had undergone liver transplantation. We excluded pediatric patients and patients who had previously undergone liver transplantation.

The following data were collected retrospectively: demographic data, etiologies of liver disease, clinical variables, donor type, intraoperative blood loss, anesthesia time, length of ICU stay and hospitalization, and outcome. The Child-Pugh points, MELD score, SOFA score, and RIFLE criteria were used to assess illness severity on the first day of admission before transplantation and on post-transplantation days 1, 3, 7 and 14. The primary study outcomes were 1-year mortality rates after liver transplantation. Follow-up at 1 year after transplantation was performed via telephone interview or by analyzing the chart records.

### Definitions

The severity of the liver disease on admission to the ICU was determined by using the Child–Pugh points and the MELD scoring systems. The MELD score was calculated with the following formula: [11].

MELD score = (0.957 ln[creatinine]+0.378 ln[bilirubin]+1.120 ln[international normalized ratio of prothrombin]+0.643)×10.

Severity of the illness can also be assessed by using the SOFA score, the CLIF-SOFA score, and the CLIF-C OF score (the CLIF-Consortium Organ Failure score, a simplified version of the CLIF-SOFA Score) based on 6 organ systems [12] (Table 1). The worst physiological and biochemical values determined on the first day of ICU admission were recorded. The RIFLE criteria were also used to group patients according to risk, injury, and failure. [13] No patient met the criteria for loss or end-stage renal disease. The following simple model for mortality was constructed: non–acute renal failure (0 points), RIFLE-R (1 point), RIFLE-I (2 points), and RIFLE-F (3 points) [14].

### Statistical analysis

Continuous variables were summarized with means and standard derivations unless otherwise stated. All variables were tested for normal distribution with the Kolmogorov–Smirnov test. Student’s t-test was employed to compare the means of continuous variables and normally distributed data; otherwise, the Mann–Whitney U test was employed. Categorical data were tested using the chi-square test. Cumulative survival curves as a function of time were constructed with the Kaplan-Meier approach and compared with the log rank test.

Calibration was assessed by the Hosmer–Lemeshow goodness-of-fit test (C statistic) to compare the number of observed and predicted deaths in risk groups for the entire range of death probabilities. Discrimination was examined using the area under the receiver operating characteristic curve (AUROC). An AUROC close to 0.5 indicates that the model performance approximates that of flipping a coin. However, the model nears 100% sensitivity and specificity despite any cutoff point as the area nears 1.0. To compare the areas under the two resulting AUROC curves we used a nonparametric approach. AUROC analysis was also performed to calculate the sensitivity, specificity, and overall correctness of the Child–Pugh points, the MELD score, the RIFLE classification, the SOFA score, and the CLIF-SOFA score. Finally, cutoff points were calculated by obtaining the best Youden index (sensitivity + specificity − 1). [15] The scores calculated at pre-OP, post-OP Day1, Day3, and Day7 were compared between 1-year survival and mortality groups by repeated-measurement analysis of variance (ANOVA) using the general linear model procedure. All statistical tests were two-tailed and a value of P<0.05 was considered statistically significant. Data were analyzed with the statistical package SPSS 12.0 for Windows 95 (SPSS, Inc., Chicago, IL, USA).

## Results

### Patient characteristics

We enrolled 323 patients who underwent liver transplantation between October 2002 and December 2010. The overall 3-month and 1-year survival rates were 86.4% (279/323) and 78.3% (253/323), respectively. Patient data and clinical characteristics of survivors and non-survivors according to in-hospital mortality are listed in Table 2. The median age of the patients was 51 years; 231 patients were men (71%) and 92 were women (29%). The median length of ICU stay was 21 days.

**Table 2:** Patient demographic data and clinical Characteristics according to In-hospital mortality.

	All patients (n = 323)	Survivors (n = 281)	Non-survivors (n = 42)	P-value
Age (years)	51±10	51±10	50±14	NS (0.187)
Gender (M/F) (%)	231(72)/92(28)	199(71)/82(29)	32(76)/10(24)	NS (0.583)
BMI (kg/m^2^)	24.3±4.0	24.7±4.0	21.1±2.4	<0.001
Diabetes mellitus (yes/no) (%)	55(17)/268(83)	46(16)/235(84)	9(21)/33(79)	NS (0.387)
Chronic kidney disease (yes/no) (%)	31(10)/292(90)	22(8)/259(92)	9(21)/33(79)	0.005
Proteinuira on admission (yes/no (%))	45(14)/278(86)	31(11)/250(89)	14(33)/28(67)	<0.001
Variceal bleeding on admission (yes/no) (%)	62(19)/261(81)	50(18)/231(82)	12(29)/30(71)	NS (0.613)
Hemoglobin on admission (g/dL)	10.6±2	10.7±2	9.8±2	0.008
Leukocytes on admission (×10^9^/L)	2.9±3.7	2.8±3.5	3.3±4.9	NS (0.569)
Platelets on admission (×10^9^/L)	73±46	73±46	71±45	NS (0.809)
Prothrombin time INR on admission	1.8±0.7	1.8±0.7	1.9±0.7	NS (0.050)
Serum sodium on admission (mmol/L)	142±69	142±74	137±8	NS (0.650)
AST on admission (U/L)	89±94	87±79	98±168	NS (0.498)
ALT on admission (U/L)	67±120	67±121	66±118	NS (0.938)
Total bilirubin on admission (mg/dL)	8.5±11.9	7.6±10.8	14.3±16.5	0.003
Lactate on admission (mmol/L)	2.1±0.8	1.5±0.8	2.9±0.9	NS (0.064)
A-a gradient on admission	251±413	233±407	316±430	0.039
Urea on admission (mmol/L)	8.3±10.3	7.8±10.7	10.1±8.82	0.007
Serum creatinine on admission (mg/dL)	1.1±1.0	1.1±1.0	1.3±1.1	NS (0.064)
MAP on admission (mmHg)	86±12	86±13	85±10	NS (0.427)
Child-Pugh points on admission	10±3	10±3	11±2	0.010
MELD score on admission	17±10	17±10	21±10	0.025
RIFLE on admission (No AKI/Risk/Injury/Failure)	286/16/9/12	250/13/9/9	36/3/0/3	NS (0.449)
SOFA on admission	5±3	5±2	7±3	0.001
CLIF-SOFA on admission	6±3	5±3	8±4	0.001
Anesthesia time (hours)	12±2	12±2	12±2	NS (0.362)
Donor type (deceased/splint/living)	51/40/232	42/32/207	9/8/25	NS (0.091)
Length of ICU stay (days)	21±23	19±22	34±27	0.002
Length of hospital stay (days)	48±32	47±30	55±39	NS (0.215)
Graft-to-recipient weight ratio (%)	1.04±0.30	1.03±0.26	1.10±0.44	NS (0.125)
Blood loss volume (ml)	3034±3731	2672±3057	4430±5431	0.014
Reimplantation time	42±11	42±11	43±11	NS (0.801)

*Abbreviations: INR, international normalized ratio; AST: aspartate aminotransferase; ALT: alanine aminotransferase; MAP, mean arterial pressure; MELD: model for end-stage liver disease; SOFA: sequential organ failure assessment; CLIF-SOFA: chronic liver failure - sequential organ failure assessment; RIFLE: the risk of renal failure, injury to the kidney, Failure of kidney function, loss of kidney function, and end-stage renal failure; ICU: intensive care unit.

The pre-transplant Child-Pugh points, MELD, SOFA, and CLIF-SOFA scores were statistically significant predictors of in-hospital mortality; the pre-transplant.

RIFLE criteria was not. Fifty-one patients (15.8%) received deceased-donor grafts; there was no significant difference in the age or gender between the survivors and non-survivors. The primary liver diseases are listed in Table 3. In this study, hepatitis B virus infection was observed to be the cause of liver diseases in most of the patients

**Table 3:** Primary liver disease.

Primary liver disease	All patients (n = 323)
Alcoholic, n (%)	47 (14)
Hepatitis B, n (%)	200 (62)
Hepatitis C, n (%)	84 (26)
Hepatoma, n (%)	88 (27)
**Single etiology**	
Alcoholic, n (%)	16 (5)
Hepatitis B, n (%)	111 (34)
Hepatitis C, n (%)	31 (10)
Hepatoma, n (%)	3 (1)
**Multiple etiologies**	
Alcoholic + hepatitis B, n (%)	21 (6)
Alcoholic + hepatitis C, n (%)	5 (2)
Alcoholic + hepatoma, n (%)	3 (1)
Hepatitis B + hepatitis C, n (%)	17 (5)
Hepatitis B + hepatoma, n (%)	49 (15)
Hepatitis C + hepatoma, n (%)	31 (10)
Alcoholic + hepatitis B + hepatoma	2 (1)
Other causes, n (%)*	34 (10)
**Total (Single etiology + Multiple etiologies)**	323(100)

*Biliary cirrhosis, biliary sclerosis, autoimmune hepatitis, Wilson’s disease, polycystic liver disease, drugs, and unknown causes.

### Calibration, Discrimination, and Severity of the Illness Scoring Systems

We have listed the results of goodness-of-fit as measured by the Hosmer-Lemeshow chi-square statistic denoting the predicted mortality risk, the predictive accuracy of the Child-Pugh points, MELD score, RIFLE criteria, SOFA score, and CLIF-SOFA score in predicting 1-year mortality in Table 4. The comparison between discriminatory values of the 5 scoring systems has also been included in Table 4. Based on the analysis of the AUROC curves, the discriminatory power of the CLIF-SOFA score was excellent. The AUROC curves of the CLIF-SOFA score calculated on post-transplant day 1, 3, 7, and 14 were significantly superior to those of the Child-Pugh points and RIFLE criteria. Moreover, the AUROC curves of the CLIF-SOFA score calculated on post-transplant day 1 and 7 were significantly superior to those of the MELD and SOFA score. The AUROC curves were highest for the CLIF-SOFA score on post-liver transplant day 7 for predicting 1-year mortality (0.877±0.033).

**Table 4:** Calibration and discrimination for the scoring methods used in predicting 1-year mortality.

	Calibration			Discrimination		
	Goodness-of-fit (x^2^)	df	p	AUROC±SE	95% CI	P
On admission						
Child-Pugh points	13.626	7	0.058	0.576±0.046	0.506–0.687	0.060
MELD score	5.519	8	0.701	0.580±0.050	0.482–0.678	0.119
RIFLE				0.566±0.054	0.460–0.671	0.202
SOFA	3.586	5	0.610	0.618±0.054	0.512–0.724	0.022
CLIF-SOFA	2.542	6	0.864	0.635±0.053	0.531–0.739	0.009
CLIF-C OF	23.315	3	<0.001	0.669±0.039	0.592–0.745	<0.001
Postoperative day 1						
Child-Pugh points	4.400	5	0.493	0.629±0.045	0.541–0.718	0.012
MELD score	5.960	8	0.652	0.637±0.049	0.541–0.734	0.008
RIFLE	1.341	2	0.511	0.591±0.054	0.485–0.696	0.078
SOFA	5.359	7	0.616	0.706±0.050	0.608–0.804	<0.001
CLIF-SOFA	9.516	7	0.218	0.788±0.047	0.695–0.880	<0.001
CLIF-C OF	2.316	4	0.678	0.712±0.039	0.635–0.789	<0.001
Postoperative day 3						
Child-Pugh points	1.271	5	0.938	0.714±0.044	0.627–0.801	<0.001
MELD score	9.404	8	0.309	0.733±0.048	0.639–0.827	<0.001
RIFLE	1.297	1	0.255	0.638±0.054	0.531–0.745	0.007
SOFA	9.968	6	0.126	0.769±0.048	0.625–0.813	<0.001
CLIF-SOFA	10.692	7	0.153	0.808±0.041	0.729–0.888	<0.001
CLIF-C OF	4.217	4	0.377	0.820±0.035	0.752–0.888	<0.001
Postoperative day 7						
Child-Pugh points	6.751	4	0.150	0.726±0.051	0.585–0.786	<0.001
MELD score	10.011	8	0.264	0.758±0.046	0.667–0.849	<0.001
RIFLE	11.967	2	0.003	0.656±0.054	0.550–0.761	0.002
SOFA	1.001	6	0.986	0.813±0.040	0.734–0.892	<0.001
CLIF-SOFA	7.395	7	0.389	0.877±0.033	0.813–0.941	<0.001
CLIF-C OF	6.378	3	0.095	0.850±0.033	0.785–0.915	<0.001
Postoperative day 14						
Child-Pugh points	5.710	3	0.127	0.763±0.040	0.685–0.840	<0.001
MELD score	23.453	8	0.003	0.792±0.047	0.700–0.884	<0.001
RIFLE	5.957	2	0.051	0.625±0.053	0.521–0.730	0.015
SOFA	10.075	7	0.184	0.807±0.042	0.724–0.889	<0.001
CLIF-SOFA	15.193	7	0.034	0.853±0.033	0.788–0.918	<0.001
CLIF-C OF	1.266	3	0.737	0.815±0.038	0.740–0.889	<0.001

*Abbreviations: CLIF-C OF: chronic liver failure-consortium organ failure; CLIF-SOFA: chronic liver failure - sequential organ failure assessment; MELD: model for end-stage liver disease; RIFLE: the risk of renal failure, injury to the kidney, Failure of kidney function, loss of kidney function, and end-stage renal failure; SOFA: sequential organ failure assessment.

### Indices for predicting short-term prognosis

**Figure 1:**
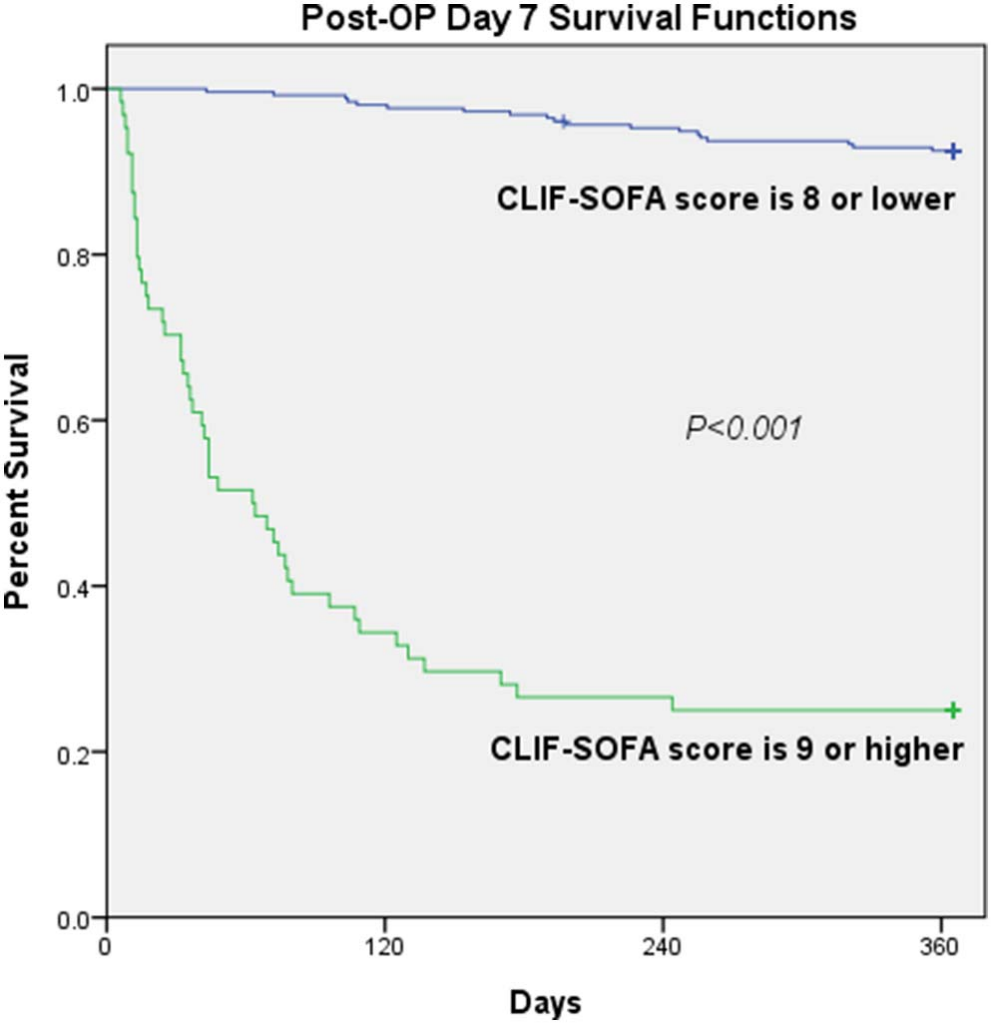
Cumulative survival rate for 323 liver transplant patients according to the CLIF-SOFA scores on day 7 after liver transplantation. *Abbreviations: CLIF-SOFA: chronic liver failure - sequential organ failure assessment.

**Figure 2:**
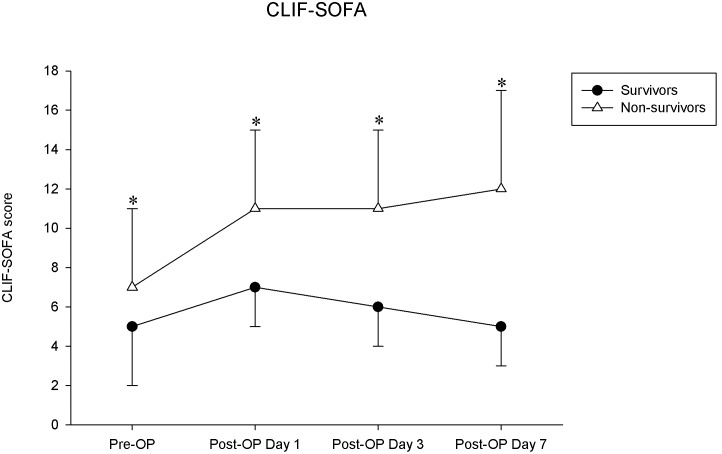
Estimated CLIF-SOFA scores (mean ± standard deviation) for the 1-year survivor group (alive, n = 253) and the 1-year non-survivor group (death, n = 70) during the preoperative period and on postoperative days 1, 3, and 7 (*P<0.05 for survivor group and non-survivor group). By repeated-measures analysis of variance, the CLIF-SOFA scores significantly increased between the period (before transplantation and on postoperative days 1, 3, and 7) in the 1-year non-survivor group but not in the 1-year survivor group. *Abbreviations: CLIF-SOFA: chronic liver failure - sequential organ failure assessment.

To assess the validity of the scoring methods, we tested the sensitivity, specificity, and overall correctness of prediction at cut-off points that provided the best Youden index (Table 5). On post-liver transplant day 7, the Youden index and overall correctness for predicting 1-year mortality were higher for the CLIF-SOFA score than those for the Child-Pugh points, MELD score, RIFLE criteria, and SOFA score. Figure 1 illustrates that the cumulative survival rates differed significantly for patients with a CLIF-SOFA score ≤8 and for those with a CLIF-SOFA score >8 on post-liver transplant day 7. Figure 2 shows significant increases in the CLIF-SOFA scores between the periods for the 1-year mortality group but not for the 1-year survival group by repeated-measures analysis of variance.

**Table 5:** Prediction of subsequent 1-year mortality.

Predictive factors	Cutoff point	Youden index	Sensitivity (%)	Specificity (%)	Overall correctness (%)
Child-Pugh points					
On admission	10	0.15	69	46	58
Postoperative day 1	10	0.25	92	34	63
Postoperative day 3	8	0.37	59	77	67
Postoperative day 7	8	0.37	51	85	68
Postoperative day 14	8	0.33	38	94	66
MELD score					
On admission	10	0.18	85	34	60
Postoperative day 1	22	0.25	85	40	63
Postoperative day 3	20	0.41	62	80	71
Postoperative day 7	20	0.43	59	84	72
Postoperative day 14	20	0.50	64	85	75
SOFA					
On admission	5	0.21	46	75	61
Postoperative day 1	9	0.37	69	68	69
Postoperative day 3	7	0.41	74	74	74
Postoperative day 7	7	0.53	67	82	75
Postoperative day 14	7	0.53	56	93	75
CLIF-SOFA					
On admission	5	0.23	59	64	62
Postoperative day 1	8	0.51	72	79	76
Postoperative day 3	8	0.54	67	87	77
Postoperative day 7	8	0.59	64	95	80
Postoperative day 14	8	0.58	67	88	78
CLIF-C OF					
On admission	6	0.35	76	59	68
Postoperative day 1	8	0.34	43	77	60
Postoperative day 3	8	0.56	78	82	80
Postoperative day 7	8	0.59	69	91	80
Postoperative day 14	8	0.53	76	78	77
RIFLE					
On admission	R category	0.13	23	90	57
Postoperative day 1	R category	0.16	36	80	58
Postoperative day 3	R category	0.24	31	94	63
Postoperative day 7	R category	0.28	46	82	64
Postoperative day 14	R category	0.22	46	76	61

*Abbreviations: CLIF-C OF: chronic liver failure-consortium organ failure; CLIF-SOFA: chronic liver failure - sequential organ failure assessment; MELD: model for end-stage liver disease; RIFLE: the risk of renal failure, injury to the kidney, Failure of kidney function, loss of kidney function, and end-stage renal failure; SOFA: sequential organ failure assessment.

### Data not shown

Only the pre-transplant SOFA score and CLIF-SOFA score were statistically significant predictors of 1-year post-transplant mortality; the pre-transplant Child-Pugh points, MELD score, and RIFLE criteria were not.

In the study population, 64 patients with CLIF-SOFA score >8 while 254 patients with CLIF-SOFA score ≤8 on day 7 post-transplantation. The patients with CLIF-SOFA score >8 on day 7 post-transplantation had higher rates of acute rejection (29.7% *vs.* 12.6%, *p* = 0.002), hospital death (51.6% *vs.* 15.0%, *p*<0.001) and 1-year mortality (75.0% *vs.* 7.5%, *p*<0.001) than those with CLIF-SOFA score ≤8 on day 7 post-transplantation.

## Discussion

In this study, the overall 3-month and 1-year survival rates were 86.4% (279/323) and 78.3% (253/323), which is consistent with that reported previously. [9], [16], [17] We found that the SOFA score and CLIF-SOFA score on admission day were independent predictors of in-hospital mortality and 1-year mortality after liver transplantation (Table 2). Our results also show that the CLIF-SOFA score is a good scoring system for predicting patient outcome and that it has better discriminatory power than the Child-Pugh points, MELD score, RIFLE criteria, and SOFA scores (Table 4). Moreover, the CLIF-SOFA score had the best Youden index and the highest overall correctness of prediction (Table 5).

Several studies had tried to find the optimal prognostic scores for critically ill cirrhotic patients. Freire P *et al* showed that SOFA and MELD scores had better overall correctness than Child-Pugh score, APACHE II, and SAPS II scores in predicting ICU mortality [18]. Levesque E *et al* reported that SOFA and SAPS II scores predicted ICU mortality better than Child-Pugh score or MELD scores with or without the incorporation of serum sodium levels [19]. Our previous studies also showed the good discriminative power and independent predictive value of the SOFA score in accurately predicting in-hospital mortality [6], [20], [21]. Since no extrahepatic parameters are included in the determination of the Child-Pugh points, and no liver-specific prognostic factors are included in the determination of the APACHE II score, their discriminative powers are significantly inferior to that of the SOFA score in predicting prognosis for critically ill cirrhotic patients. The prognosis of cirrhotic patients is grave and liver transplantation is the treatment of choice. Liver transplantation improves survival rate of patients with end-stage liver disease dramatically therefore impacts the capability of pre-transplant scoring systems in predicting short-term prognosis of post-transplant patients.

Theocharidou E *et al* had proposed the Royal Free Hospital (RFH) Score from a cohort of 635 critically ill cirrhotic patients, which included variceal bleeding, bilirubin, INR, lactate, A-a gradient and urea. The AUROC of the pre-transplant RFH score is 0.600 in predicting 1-year survival for liver transplantation patients in this study, it is even inferior to that of the pre-transplant SOFA (AUROC = 0.618) and CLIF-SOFA (AUROC = 0.635) scores. Based on our clinical experience, we think the 6 parameters of the RFH score are good predictors in predicting short-term prognosis for patients with portal hypertension. However, liver transplantation dramatically turns the course of disease in decompensated cirrhotic patients and post-OP critical care is the key for post- transplant patient survival. Other mortality risk factors are technical problems (especially vascular and biliary anastomoses), rejection, primary graft failure, opportunistic infection, and drug reaction. CLIF-SOFA and SOFA scores could evaluate parameters related to 6 different important organ systems and provide a global assessment of the patient’s clinical condition. It might explain the good prediction value of the CLIF-SOFA and SOFA scores. For lacking of CNS and CV parameters, the performance of RFH score is slightly inferior to that of the SOFA and CLIF-SOFA scores in predicting short-term prognosis for patients undergoing liver transplantation.

Similar to other general ICU scores, the SOFA score was developed for the general ICU population. Many studies have reported that the SOFA score could provide a complete representation of illness dynamics, and patients with a higher SOFA score are associated with a lower probability of receiving liver transplantation. [22], [23] However, it is possible that some components of the SOFA score could be influenced by the nature of liver disease. For example, platelet counts are always reduced in cirrhotic patients due to hypersplenism, reduced production of thrombopoeitin, alcohol consumption, or antiviral treatment. [24], [25] Relatedly, no association has been reported between low platelet level and outcome of cirrhotic patients. [24]–[26] The CLIF-SOFA score is a newly developed scoring system that is a modified version of the SOFA score (Table 1), and that is exclusively for patients with end-stage liver disease. It replaces platelet count with an international ratio of prothrombin time as the coagulation parameter, and replaces the Glasgow coma scale with hepatoencephalopathy as the CNS parameter. It also takes into account the usage of terlipressin and renal replacement therapy in the grading of cardiovascular and renal parameters, respectively. Furthermore, the CLIF-SOFA score added SpO2/FiO2 as an alternative respiration parameter for patients without an A-line. All these modifications were set up especially targeting the disease nature and general treatment protocol of end-stage liver disease [10]. In this study, although both pre-transplant SOFA score and CLIF-SOFA score were statistically significant predictors of 1-year post-transplant mortality, the discriminatory power of CLIF-SOFA score was even superior to that of the SOFA score on post-transplant day 1, 3, 7, and 14 (p<0.05 on post-transplant day 1 and day 7). Both SOFA score and CLIF-SOFA score provided a complete representation of illness dynamics in serial assessment before and after transplantation, but the CLIF-SOFA score showed greater numerical differences between the 1-year survivor group and non-survivor group, especially during the post-transplantation period (Figure 2). Moreover, trends in the CLIF-SOFA score reflect a patient’s response to therapeutic strategies, [9], [23], [27] with a CLIF-SOFA score >8 on post-transplant day 7 indicating a delayed recovery of multiple organ dysfunction from operation that is associated with a higher rate of acute rejection and poor 1-year survival rate (Figures 1–2). Because of implications for graft survival, the diagnosis of acute rejection and its prompt treatment is very important for these patients.

Recentlly, Jalan *et al* from the CLIF Consortium have generated a simplified version of the CLIF-SOFA Score (the CLIF-Consortium Organ Failure score, CLIF-C OFs, which has only 3-point range per organ system) [12] (Table 1). The performance of the CLIF-C OF score is similar to that of the CLIF-SOFA score and superior to that of the SOFA score significantly (Table 4–5). It is also an excellent scoring system in predicting short-term prognosis for liver transplantation patients. In the same study, Jalan *et al* also elaborated a specific score for patients with acute-on-chronic liver failure (CLIF-Consortium score for ACLF, CLIF-C ACLFs) that includes the CLIF-C OFs plus age and white-cell count. The accuracy of the CLIF-ACLF score is even superior to that of the CLIF-SOFA and CLIF-C OF scores in the study of Jalan *et al*. However, the performance of the CLIF-ACLIF is inferior to that of the CLIF-SOFA score in this study (data not shown). There are some explanations for the discrepancy of the study results. First, in this study, age is not significantly associated with in-hospital mortality rate (table 2) and this finding is consistent with our previous reports [6], [20], [21]. Hepatitis B virus-related liver cirrhosis is the major population in our country, while alcoholic cirrhosis is the major population in Europe. The difference of prediction value of age might be attributed to the different population between our studies and European ones. Second, the usage of prednisolone and other immunosuppressant might impact the application of white blood cell count in predicting outcome for liver transplantation patients. Above 2 reasons might, at least partially, explain why the CLIF-C ACLF score is not an optimal score in predicting prognosis for patients undergoing liver transplantation in our study. Another well-powered trial is required to examine this issue.

In spite of the encouraging results observed in our study, several potential limitations should be recognized. First, the fact that our study was conducted at a single tertiary medical center limits the generalization of the findings to other hospitals with different patient populations. Second, because of the retrospective nature of this investigation, some clinical variables were unavailable. Third, in our study, given that hepatitis B viral infection was the leading cause of liver cirrhosis, the use of our classification system may not be appropriate for patients in North America and in Europe where liver diseases are mostly attributed to hepatitis C viral infection and alcoholism. The patient population contained a high proportion of hepatitis B (62%) patients and hepatoma (27%) patients (Table 3), and may present as a special subgroup in the cirrhotic patient. Finally, the predictive accuracy of logistic regression models had its own limitations.

## Conclusion

In conclusion, the short-term prognosis after liver transplantation is best predicted by the CLIF-SOFA score. Our data suggest that the SOFA and CLIF-SOFA scoring systems were independent predictors of 1-year mortality after liver transplantation. The analytical data also showed the CLIF-SOFA score is superior to the Child-Pugh points, MELD score, RIFLE criteria, and SOFA score in predicting short-term prognosis. We confirmed that the pre-transplant and post-transplant CLIF-SOFA scores are accurate and capable of providing an improved prediction of prognosis along with objective information for clinical decision making for treating this subset of patients. On the basis of the observed results, we recommend that a CLIF-SOFA score >8 on post-transplantation day 7 be considered as high risk of acute rejection and negative short-term outcome. Graft biopsy is suggested for these patients to diagnosis and to guide antirejection therapy.
